# Types and Functions of Mitoribosome-Specific Ribosomal Proteins across Eukaryotes

**DOI:** 10.3390/ijms23073474

**Published:** 2022-03-23

**Authors:** Vassilis Scaltsoyiannes, Nicolas Corre, Florent Waltz, Philippe Giegé

**Affiliations:** 1 CNRS, Institut de Biologie Moléculaire des Plantes, Université de Strasbourg, 67084 Strasbourg, France; vasileios.skaltsogiannis@etu.unistra.fr (V.S.); nicolas.corre@etu.unistra.fr (N.C.); 2 Helmholtz Pioneer Campus, Helmholtz Zentrum München, Ingolstädter Landstraße 1, 85764 Munich, Germany

**Keywords:** mitochondrial gene expression, ribosomes, translation, helical repeat proteins, pentatricopeptide repeat proteins, single particle cryo-EM

## Abstract

Mitochondria are key organelles that combine features inherited from their bacterial endosymbiotic ancestor with traits that arose during eukaryote evolution. These energy producing organelles have retained a genome and fully functional gene expression machineries including specific ribosomes. Recent advances in cryo-electron microscopy have enabled the characterization of a fast-growing number of the low abundant membrane-bound mitochondrial ribosomes. Surprisingly, mitoribosomes were found to be extremely diverse both in terms of structure and composition. Still, all of them drastically increased their number of ribosomal proteins. Interestingly, among the more than 130 novel ribosomal proteins identified to date in mitochondria, most of them are composed of a-helices. Many of them belong to the nuclear encoded super family of helical repeat proteins. Here we review the diversity of functions and the mode of action held by the novel mitoribosome proteins and discuss why these proteins that share similar helical folds were independently recruited by mitoribosomes during evolution in independent eukaryote clades.

## 1. Introduction

Ribosomes are key molecular machines, fundamental to all life on Earth. They decode information carried by messenger RNAs (mRNAs) and translate it into proteins [1]. Ribosomes are universally composed of two subunits, the small one (SSU) binds mRNA and reads the genetic information, while the large subunit (LSU) catalyses the actual formation of peptidyl bonds between amino-acids that allows protein synthesis. Likewise, all ribosomes are ribonucleoprotein (RNP) complexes composed of numerous proteins and ribosomal RNAs (rRNAs), the LSU rRNA being a catalytic RNA, i.e., a ribozyme. Despite these common features, ribosomes diverged significantly during evolution. In bacteria and archaea, ribosomes both make 70S particles with about 54 proteins as well as 16S, 23S and 5S rRNAs [2]. However, archaeal ribosomes often evolved to adapt to harsh environmental conditions e.g., with the recruitment of specific r-proteins to respond to high temperature or halophilic conditions. Thus archaeal ribosomes often have a more rigid structure than their bacterial counterparts [3]. In contrast, ribosomes that occur in eukaryote cytosol are larger, forming 80S RNP complexes. They evolved from an ancestral archaeal ribosome but have more proteins and larger rRNAs, e.g., with 80 proteins and 18S, 28S, 5S and 5.8S rRNAs in humans [4]. Beyond these cytosolic ribosomes, which are often referred to as “eukaryote ribosomes”, other types of ribosomes occur in eukaryote cells. These additional ribosomes are found in genome-containing organelles: mitochondria, as well as chloroplasts for the Archaeplastida kingdom. Until the last decade, very little was known of these organellar ribosomes [5,6].

Mitochondria and chloroplasts are essential energy producing organelles of bacterial origin that have arisen through distinct endosymbiotic events. Mitochondria derive from a bacteria belonging to a sister group of α-proteobacteria that was engulfed by the archaeal-type ancestor of eukaryote cells about 1.5–2 billion years ago [7,8,9,10]. The second endosymbiotic event took place 500 million years later with the engulfment of a cyanobacteria by a fully ledged eukaryote that gave rise to the tripartite phylum (red, blue and green algae) Archaeplastida [11,12]. Both mitochondria and chloroplasts are considered as semi-autonomous organelles because they have retained a genome and fully functional gene expression machineries [5]. While the vast majority of bacterial genes coming from the ancestral endosymbionts were either lost or transferred to the host nuclei, some genes—most of them encoding essential proteins of the respiratory chain or the photosynthetic machinery—remain to this date in organellar genomes. It is notable that many of these genes encode hydrophobic core proteins of the respective energy producing systems. The difficulty to synthesize in the cytosol and import to the organelles such membrane proteins might have been an evolutionary indicator for the retention of their genes in organellar genomes [13]. Another hypothesis proposes that a colocation of genes and of their products is required for the redox regulation of gene expression [14]. Nonetheless, the retention of mRNA coding genes in organelles implied that comprehensive gene expression machineries, including in particular functional ribosomes, should be present in organelles. For their biogenesis, mitochondrial ribosomes thus rely on the coordinated synthesis of ribosomal proteins, most of whose mRNAs are coded in the nucleus, translated in the cytosol, and imported into mitochondria, together with the synthesis of moieties encoded by the mitochondrial genome, i.e., mitochondrial rRNAs and ribosomal proteins, in some eukaryotes. Few studies have investigated the mitoribosomes assembly, although the evidence suggests that, in human and yeast, this process resembles bacterial ribosome assembly [5]. Because of their bacterial origin, it was assumed for a long time that organellar ribosomes would be very similar to bacterial ones. This assumption turned out to be true for chloroplasts, where the chlororibosomes composition and three-dimensional structure strongly resemble those of bacterial ribosomes, as revealed by recent studies [15,16,17,18].

**Figure 1:**
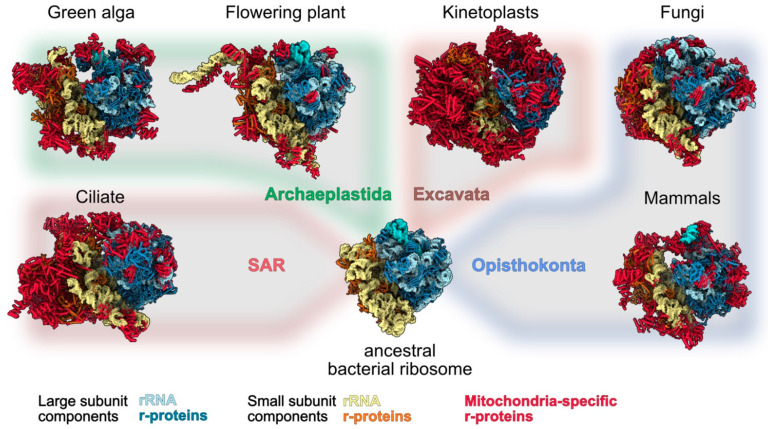
Structural comparison between available mitoribosome structures. To highlight the divergence of mitoribosomes as compared to bacteria and their diversity in terms of size and shape across eukaryotes, high resolution structures of mitoribosomes are presented and overlaid on a schematic evolutionary tree of eukaryotes. From left to right, mitoribosomes structures are from: ciliate (*T. thermophilus*), green alga (*C. reinhardtii*), flowering plant (*A. thaliana*), kinetoplasts (*T. brucei*), fungi (*N. crassa*) and mammals (*H. sapiens*). They are compared with *E. coli* ribosome structure, representing the ancestral form of mitoribosomes. Large subunits components are shown in blue shades and small subunit components in yellow shades. Mitochondria-specific ribosomal proteins (which includes shared and species-specific proteins) are highlighted in red. No structures have been solved yet for the supergroup Amoebozoa (hence not shown on the figure). SAR corresponds to the supergroup which includes the Stramenopiles, Alveolates, and Rhizaria subgroups.

**Figure 2:**
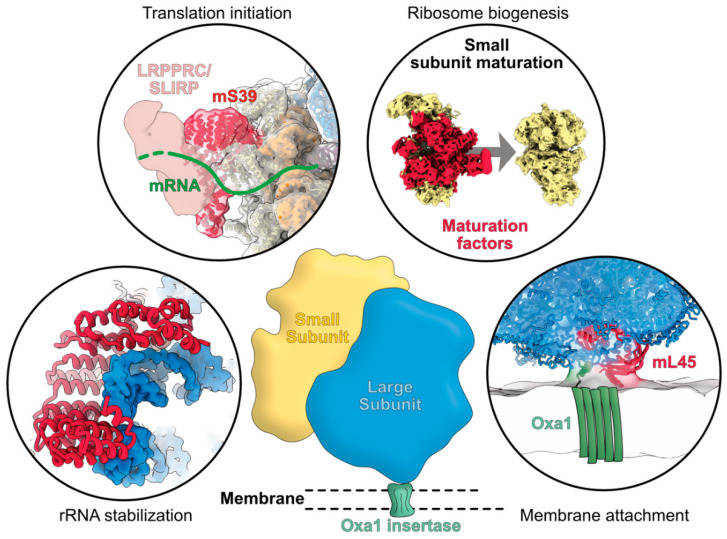
Graphical summary of the functions of mitoribosome-specific ribosomal proteins. The recently identified proteins specifically occurring in mitochondrial ribosomes are shown in red. They can be involved in rRNA stabilization, in the recruitment of mRNA for translation initiation, in the assembly and maturation of mitoribosomes, and in its attachment to the mitochondrial inner membrane through the binding with the insertase Oxa1. The exemplary shown mS39 and mL45 specifically occur in mammalian mitoribosomes.

In marked contrast with chloroplasts, the recent characterization of mitochondrial ribosomes (mitoribosomes) in a diversity of species representing the major eukaryote groups has revealed how mitoribosomes strikingly diverged from prokaryotes and eukaryotes ribosomes. Even more stunning is their diversity between the different eukaryote clades [5,6]. While the 3D structures of bacterial and cytosolic ribosomes could be obtained by X-ray crystallography in 1999 and 2011, respectively [19,20], the characterisation and determination of 3D structures from very low abundant ribosomes such as mitoribosomes was only possible thanks to the cryo-electron microscopy (cryo-EM) revolution that started a decade ago. Since 2015, the compositions and structures of ribosomes from animals, fungi, kinetoplasts, land plants, a ciliate and a photosynthetic algae could be determined (Figure 1) [21,22,23,24,25,26,27,28], thus providing examples of mitoribosomes from Opisthokonta, Excavata, Archaeplastida and Stramenopiles, Alveolata, and Rhizaria (SAR), four out of the five major super-groups of eukaryotes. The overall architecture of all these mitoribosomes is extremely diverse, in particular that of the SSUs with numerous specific rRNA expansion segments and additional domains [5,29]. Mitochondrial ribosomal RNAs are extremely diverse. They can be larger than in bacteria, as seen in yeast and particularly in land plants, or highly reduced, as observed in animals and particularly in kinetoplasts. The most extreme example of rRNA divergence was observed in the algae Chlamydomonas, where rRNAs are fragmented into 13 pieces that are non-contiguously encoded as gene pieces in the Chlamydomonas mitochondrial genome, expressed as separate small RNAs and brought together to reconstitute a functional ribosome [27]. Beyond these divergences, a common feature of all mitoribosomes is the marked increase in the number of ribosomal proteins. To date, over 130 novel ribosomal proteins, specific to mitoribosomes, have been identified, although only about 30 novel proteins are common to at least two mitoribosomes [30]. This implies that beyond a set of common proteins that might have been present in the common ancestor of all mitoribosomes, the different eukaryote groups independently recruited specific sets of proteins to make specialised ribosomes, potentially adapted to different environmental niches. Still, despite this diversity, it is remarkable that most of the novel mitochondrial ribosomal proteins belong to the group of a-helical repeat proteins, including proteins such as pentatricopeptide repeat (PPR) proteins, octotricopeptide repeat (OPR) proteins or mitochondrial transcription termination factor (mTERF) proteins that share a similar type of tridimensional fold. The diversity of structures and functions of the novel mitochondrial ribosomal proteins, for rRNA stabilisation, for the recruitment of mRNA, to bind other ribosomal proteins, to anchor the ribosomes to the mitochondrial membrane and for the assembly of mitoribosomes will be discussed here, and insights will be provided to understand the diversity of their mode of action. This ensemble of functions is summarized in Figure 2.

## 2. Technologies That Allowed to Identify Mitoribosome-Specific Proteins

As early as the late 1950s, it was speculated that complexes capable of protein synthesis would be present in mitochondria [31,32], only five years after the first description of ribosomes [33]. In the following 10 years, biochemical characterisation of mitoribosomes was achieved for fungal and animal species [34]. These early reports described the sedimentation coefficient and the probable rRNA content of these mitoribosomes, already linking them to bacteria. However, the clarification of their protein composition would also only come more than 10 years later, with the two-dimensional poly acrylamide gel electrophoresis (PAGE) analysis of purified bovine mitoribosomes, revealing that these mitoribosomes would be composed of more than 80 proteins, hence more than found in bacteria, yet without knowing exactly their nature [35]. The development of protein sequencing and mass-spectrometry in the 1990s allowed, in the early 2000s, for the first characterisation of the putative protein composition of the small and large subunit of the mammalian mitoribosome [36,37]. These studies already revealed the conservation and absence of bacterial homologs, as well as putative proteins shared with yeast mitoribosomes, and some being specific to animals. However, their role in the ribosome was unknown. In 2003, the first cryo-EM characterization of a mitoribosome was published [38]. This study reported the architecture of the bovine mitoribosome at about 10Å resolution, not enough for a clear assignation of the mitoribosome specific r-proteins. It confirmed, however, the largely proteic nature of this mitoribosome, looking significantly different from bacterial and cytosolic ribosomes.

The release of complete organisms genomes combined with basic local alignment search tool (BLAST) and mass-spectrometry analyses allowed to investigate the composition of mitoribosomes in new organisms, revealing conserved and specific proteins [39,40,41,42]. Still, small proteins can escape detection by mass-spectrometry, and really pure samples are required to draw a clear picture of the protein composition, otherwise leading to an overestimation of the protein content, and the mis-assignation of proteins as r-proteins. The combination of different purification techniques and analyses, such as immunoprecipitation and classical sucrose density gradient separation, or large pore Blue Native PAGE and complexome profiling, or grad-seq, provides more accurate protein composition determination [26,43,44,45] but is still not 100% reliable and does not provide functional and spatial localization of the proteins in the complex of interest. These experiments can be complemented by crosslinking mass-spectrometry which allows for the determination of what proteins or domains are close to each other in the complex [46].

More than 50 years have been necessary to achieve a clear idea of the complete composition of a mitoribosome. Structural biology elucidates both the composition and function of the studied complex. Thanks to the rapid technical advancement of cryo-EM in the 2010s, notably with the development of new direct electron detectors [47], larger and more challenging to purify complexes could be resolved, such as mitoribosomes [21,22,23,48,49], to resolutions that allowed unambiguous assignation of protein densities, thus allowing for the direct determination of the complexes’ composition.

Today, thanks to the technical progress in proteomics, metagenomic and structural biology, the combination of these techniques allows for the investigation of the composition of mitoribosomes and their evolution in ways that were unimaginable before.

## 3. Prominence of Helical Repeat Proteins in Mitochondrial Gene Expression

As mentioned above, recent technological advances, in particular cutting edge single particle cryo-EM [47], has enabled the identification of a large number of ribosomal proteins that specifically occur in mitochondria. Many of these factors belong to the protein super-family of helical repeat proteins that are composed of repeated modules of helices. The occurrence of helical repeat proteins as ribosomal proteins might not be surprising because, for two decades, a fast growing number of genomic and functional investigations have identified that many organelle specific post-transcriptional processes are performed by helical repeat proteins belonging to recently recognized gene families such as pentatricopeptide repeat (PPR), octotricopeptide repeat (OPR), mTERF, half-a-tetratricopeptide repeat (HAT) and heptatricopeptide repeat (HPR) proteins [50].

For instance, PPR proteins that occur in all eukaryotes and are particularly prevalent in plants with hundreds of PPR genes were found to be involved in the maturation of RNA ends, RNA editing, splicing, RNA stabilization and translation [51]. Interestingly a mode of action has been described for PPR proteins where specific amino-acids at given positions in individual PPR motifs give specificity for a particular ribonucleotide. Thus, a succession of PPR motifs recognizes single stranded RNA in a sequence specific manner. This connection between amino-acids of PPR motifs and nucleotides is referred to as the “PPR code” [52]. This code was validated experimentally for many proteins, i.e., for RNA editing factors [51]. However, this mode of action is not applicable to all PPR proteins, as, for example, recently recognized PPR-NYN proteins holding RNase P activity, that recognize folded pre-tRNAs in a non-canonical way [53,54,55].

HAT proteins make a smaller family of proteins, conserved in eukaryotes, where they were found to be involved in a variety of organellar but also nucleo-cytoplasmic processes [56]. For instance, the HAT protein HCF107 was found to bind and most probably stabilize the 5′ ends of chloroplast mRNAs [57].

Likewise, the mTERF protein family that occurs in plants and animals appears to be specific to organelles [58]. mTERF proteins were found to bind DNA but also RNA with, fodr example, MTERF4 that was found to bind rRNA to regulate ribosome biogenesis in humans [59], and mTERF9 that interacts with the 16S rRNA to promote chloroplast ribosome biogenesis in Arabidopsis [60].

In contrast, OPR proteins appear to be restricted to the plant lineage as well as to Alveolata, and are particularly prevalent in Chlorophyta such as Chlamydomonas. OPR proteins are related to a variety of RNA related processes in the chloroplast, for example, [61,62], and the OPR protein RAP was found to be required for chloroplast 16S rRNA maturation [63].

Finally, HPR proteins were recently recognized to be a family of putative organellar proteins which are mostly found in Alveolata. They were experimentally shown to occur in mitochondria of the malaria parasite Plasmodium, where they were proposed to be involved in RNA processing and/or stabilization [64], possibly for the assembly of the fragmented rRNAs that occur in apicomplexa mitoribosomes, similar to Chlamydomonas.

Beyond OPR and HPR that appear to be related [64], all these gene families do not seem to have a common evolutionary origin [50]. In particular, consensus motifs derived from the respective protein families do not appear to hold conserved sequence features [50]. Nonetheless, all these proteins have a similar modular organization, with tandem arrays of individual repeats that all contain a similar secondary structure relying on antiparallel a-helices. In all these different protein families, the succession of repeats folds into a solenoid structure. It is this conserved fold that assigns these organelle proteins to the super-group of helical repeat proteins that also includes PUF and TALE proteins [65,66]. Interestingly, the comparison of structure models for representative factors from the different families had suggested that many helical repeat proteins contain nucleic acid binding platforms composed of positively charged residues in the concave surface of the super-helices [50]. This feature that was proposed for PPR and mTERF proteins [59,67] has been shown experimentally for PUF proteins [68] and also observed recently for many of the ribosomal helical repeat proteins by the recent cryo-EM studies of mitoribosomes. In this light, it is tempting to speculate that helical repeat proteins might share at least some features used for RNA recognition.

To date, among helical repeat proteins, the PPR family has clearly attracted the most attention, for instance with the description of a PPR code. Interestingly, as described in details below, none of the PPR proteins identified as ribosomal proteins seem to recognize RNAs according to the PPR code. At this stage, it is unclear whether the occurrence of PPR proteins as ribosomal proteins constituted an ancestral function for these proteins and the PPR code was acquired later for specific processes or whether the mode of action using the PPR code was ancestral and subsequently lost for some functions, such as the association to ribosomes in different eukaryote groups. Future investigations will contribute to decipher the diversity of the helical repeat proteins mode of action to try to understand why this type of structure was independently selected for nucleic acid binding several times in evolution, in particular in the context of mitoribosomes.

## 4. Functions and Modes of Action of Mitoribosome-Specific Proteins

### 4.1. rRNA Stabilization Mediated by Mitoribosome-Specific Proteins

Ribosomes are complex ribonucleoprotein particles composed of rRNAs and r-proteins. Most of the r-proteins are located at the periphery of the ribosomes. These proteins are usually composed of a globular domain facing the solvent, and long domains extending into the ribosome, between rRNA helices, to anchor the protein and/or stabilize the rRNA. Even though prokaryotic and cytosolic ribosomes have now been characterized in detail, it is unclear what actual function these r-proteins possess. Given that the majority of them are largely positively charged, it is suggested that the general function of r-proteins is to stabilize and counteract the negatively charged rRNAs which perform the catalytic activity [69]. Hence, their main role is to stabilize rRNAs to promote efficient protein synthesis.

In mitoribosomes, most of the additional r-proteins that were acquired during eukaryote evolution also appear to serve this function (additional functions are described in the following sections). However, due to the diversity of rRNA composition across mitoribosomes, many of these proteins have been recruited to fit a certain need, e.g., stabilization of an rRNA extension, or compensation of rRNA loss.

Some of these proteins are conserved between mitoribosomes and were surely acquired early in their evolution. This is, for example, the case of mS29, a GTPase located on the head of the small subunit, first described in humans [21,22]. Resolution was sufficient in human, yeast and kinetoplastids to show that the GTPase do contain GDP and might be active, however its role in the mitoribosome is unclear [23,24,29]. In all cases, it binds to rRNA of the small subunit, and might be involved in subunit association and translocation by making specific bridges between the SSU and the LSU.

**Figure 3:**
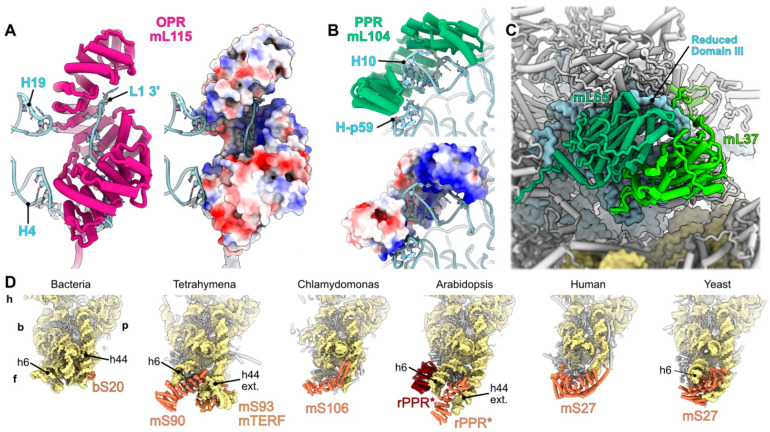
Examples of RNA-binding proteins recruited to mitoribosomes. (**A**,**B**) present close-up views of large alpha-helical proteins which were largely recruited to mitoribosomes. (**A**) The OPR mL115 involved in the stabilization of fragment L1 of the fragmented rRNAs of C. reinhardtii is shown. The OPR enlaces the 3′ single-stranded extremity of the L1 fragment which is stabilized via charge interaction. It also interacts with H19 and H4. The electrostatic potential of mL115 is shown with blue surfaces showing positive charges and red surfaces showing negative charges. (**B**) The PPR mL104 of the flowering plant mitoribosome is shown. It englobes helix H10, interacting with the rRNA backbone which is a different mode of action compared to non-ribosomal PPR proteins. It also interacts with H-p59, a plant specific RNA helix. As in (**A**), the electrostatic potential of mL115 is shown, revealing that the rRNA interaction is mostly mediated by positively charged residues. (**C**) The pseudo-dimer composed of mL65 and mL37, probably repurposed endonucleases, are shown as an example of non-alpha-helical mitoribosome-specific r-proteins. The r-proteins interact with the reduced domain III of the large subunit of the mammalian mitoribosome. (**D**) Structural comparison of the foot of the small subunit. This comparison highlights the conserved helical proteins involved in the stabilization of the foot of the small subunit—where the tip of h44 and h6 are either extended, reduced, or deleted—most likely due to the loss of the bacterial r-protein bS20 in mitoribosomes. All mitoribosomes share a PPR protein at this position (mS27, mS90, rPPR* and mS106), which may be the product of a single ancestral PPR protein. In trypanosoma, PPR protein mS63 is also present at this position but no longer interacts via RNA. The different parts of the small subunit are indicated on the bacterial model; h: head, b: body, f: foot, p: platform.

One interesting case is the systematic acquisition of PPR proteins at the foot of the small subunit (Figure 3D). In bacteria, the foot of the SSU is formed by the tip of the rRNA helix h44 and the helix h6—the only protein present in that area is bS20, which probably contributes to h44 stabilization. However, bS20 was universally lost in the mitochondria [5]. The presence of a PPR protein in the that area was first described in humans, where the protein mS27 compensates for the strong reduction and unwinding of h44 and the loss of h6 [21,22]. It was later discovered that mS27 is also present in yeast, however the protein here carries an additional function, as the rRNA is more conserved with bacteria in yeast compared to humans and is even expanded [70]. There, it stabilizes h44, h6 and the fungi expansion segment h44-ES1. In land plants, a PPR protein is positioned similar to the yeast mS27, and helps stabilize the extended h44 tip [25]. In the ciliate Tetrahymena, the rRNA in this area is significantly expanded, and two proteins were recruited to stabilize it [28]. mS93 is an mTERF protein involved in the stabilization of the expanded h44, and mS90, which resembles a PPR protein, is positioned similar to the yeast mS27 and the PPR protein found in land plants. Finally, in the green algae Chlamydomonas, where the rRNAs are reduced—h6 is noticeably absent —a PPR protein, mS106, is found, and is positioned similar to mS27 in humans [27]. Interestingly, in kinetoplastids, two PPR proteins, mS51 and mS63, are located in that area, which no longer contains RNA due to the drastic rRNA reduction in these organisms [24]. mS51 and mS63 are positioned similar to mS90 and mS93, respectively, in Tetrahymena. It is remarkable to systematically find these very similar proteins at that position in all mitoribosomes investigated to date. One explanation could be that similar proteins would have been independently recruited to perform a similar function in the different eukaryote groups. Alternatively, a PPR protein might have been recruited very early during mitoribosome evolution to compensate for bS20 loss, and have drifted significantly during evolution. This remains to be properly investigated.

rPPR proteins seem to have a totally distinct RNA binding process than the canonical PPRs, where a “PPR code” was described. In particular, in plants, no RNA targets could be predicted using the PPR code when looking at positions 5 and 35 of each rPPR protein motif, suggesting that RNA interactions are performed differently. This was confirmed by the cryo-EM structure of a plant mitoribosome. It revealed the contact sites between rPPR proteins and rRNAs [25]. It appears that most rPPRs contact double stranded RNA helices of the rRNA instead of single stranded RNA (Figure 3B). This interaction is mediated by positively charged residues of the rPPRs and the negatively charged phosphate backbone of the rRNAs. How this process achieves the specific binding of individual rPPR proteins to their interaction site on the mitoribosome is still undetermined.

In the case of specific proteins being recruited, in Chlamydomonas, where rRNAs are reduced and fragmented, several OPR proteins were recruited to the ribosome, and help stabilize the extremities of the rRNA fragments [27]. They mainly interact with single stranded rRNAs by enlacing the rRNA ends, but can also interact with rRNA helices via their solvent exposed side. This is the case for mL115, which stabilizes the L1 rRNA 3′ end, and also interacts with H4 and H19 of the same rRNA fragment (Figure 3A). A similar case is found in the ciliate Tetrahymena, where the mL106 protein is also involved in stabilizing the 3′ end of one of the two rRNAs of the large subunit [28].

These RNA/protein interactions are reminiscent of the canonical ssRNA/PPR interactions, although no recognition code between OPR proteins and single stranded RNA could be recognized at this stage. In contrast, mS107, an OPR from the SSU of Chlamydomonas, binds the double stranded RNA of the S2 rRNA, similar to the land plant mitoribosome rPPRs. mL113, an OPR protein, binds the tip of a helix from L3b thanks to a domain inserted between OPR repeats; in that case OPR repeats are not at all involved in RNA binding.

In animals, specific proteins, a pseudo-dimer made by mL65 and mL37, repurposed endonucleases, interacts with the reduced domain III of the large subunit. There, it probably stabilizes the rRNA and compensates for its loss (Figure 3C) [21,22]. In Chlamydomonas, the strong reduction of domain III is compensated for by the acquisition of the OPR mL113 and the mTERF mL114 [27]. As opposed to these cases, rRNAs are expanded in flowering plants. In the large subunit, the rPPR proteins mL101 and mL104 are involved in the stabilisation of the refolded and extended domain III and domain I. mL104, for example, englobes the tip of H10 from domain I and interacts with H-p59, a helix from the refolded domain III (Figure 3B) [25].

Altogether, mitoribosomes have recruited a wide variety of binders that bind their RNA targets through very diverse processes.

### 4.2. Mitochondrial Specific mRNA Recruitment Processes

**Figure 4:**
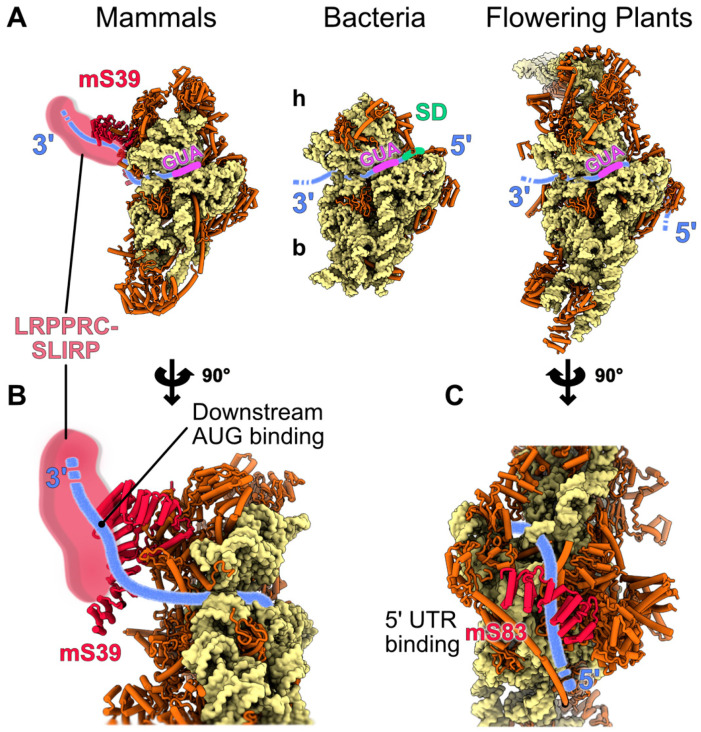
Specific proteins involved in the translation initiation process in mitochondria. Proposed mechanisms of mitochondria-specific translation initiation events involving mitochondria-specific r-proteins are shown. (**A**) The proposed positions and orientations of the mRNA on the small subunits of mammalian, plant and bacteria during translation initiation are indicated on the figure. The mRNA is shown in blue, the initiation codon (AUG) is in purple and the Shine-Dalgarno (SD) consensus, only present in bacteria, is in green. (**B**,**C**) present close-up views of mammalian and plant mRNA binders. (**B**) The mammalian mRNA stabilization by the PPR protein mS39 and probably the LRPPRC-SLIRP complex is shown. mS39 and LRPPRC-SLIRP interact with the ORF, downstream of the AUG. (**C**) In flowering plants, a proposed mechanism of mRNA stabilization would involve the PPR protein mS83, which would bind a consensus sequence located in the 5′ UTR of the mitochondrial mRNAs. A similar process was also proposed in yeast, involving specific proteins for each yeast mRNA.

During translation initiation, the mRNA is recruited to the SSU, where its start codon is aligned with the ribosomal P site and recognized by the initiator tRNA. This first step of the translation cycle exhibits a great variation across lineages and has attracted a lot of interest in the last decades. In bacteria, the mRNA binding to the ribosome is often directed by an interaction between a conserved sequence motif, the Shine-Dalgarno (SD), and a complementary sequence, the anti Shine-Dalgarno, located at the 16S rRNA [71,72,73]. The SD motif is positioned upstream of the start codon and aids in aligning the ribosome to select and decode the open reading frame (Figure 4A). This Shine-Dalgarno dependent mechanism is the most widely used for prokaryotic mRNAs, but other mechanisms involve, for example, the S1 protein [74] and some mRNAs that are completely devoid of SD sequence, or even 5′UTR [75]. For the later, it remains elusive how they are recruited to the ribosome [71]. Thus, mRNAs with discrete characteristics require distinct mechanisms for their recruitment.

In mitochondria, despite the plethora of proteins that partake in their proteome, only a few are encoded by the mitochondrial expression machinery, i.e., 13 in human, eight in yeast [6], and around 30 in land plants [76,77], where the majority are components of the oxidative phosphorylation complexes. In most eukaryotes, mitochondria-encoded mRNAs lack the SD sequence and the 5′ m7G cap, features that aid their translation in bacteria and cytosol, respectively, the exception being some Jakobid species such as Reclinomonas, which retained SD-like sequences [78]. Moreover, the 5′ untranslated region (5′UTR), which contains cis-elements essential for expression, varies in length across lineages and in some cases is missing. The heterogeneity that mitochondrial mRNAs present, coupled with the profound divergence of mitoribosomes structure and composition across various phyla, have resulted in specialized recruitment mechanisms. For instance in mammals, most mitochondrial mRNAs do not possess a 5′ UTR and when they do, it is up to 3 nt in length [79,80,81], leaving little, if any space for a 5′ UTR-dependent regulation. In compliance with this notion, a specific ribosomal protein has been proposed to direct mRNA binding in humans. The PPR protein mS39 is located at the entry site of the mRNA channel on the ribosomal small subunit [21,22]. Allegedly, mS39 PPR motifs interact with a U-rich region placed downstream of the start codon in the vicinity of the mRNA’s 7th codon, facilitating the recruitment of the mRNA to the ribosome [82]. Moreover, a recent study revealed an additional moiety, the leucine-rich PPR motif-containing protein (LRPPRC), forming a complex with the Stem-Loop Interacting RNA binding Protein (SLIRP), that interacts with mS39 in translationally active mitoribosomes, i.e., bound mRNA and tRNA present in A or P site [83]. The LRPPRC-SLIRP complex shows a strong affinity for RNAs via several PPR motifs [84], promotes mRNA polyadenylation in mitochondria [85], and acts as a chaperon to mediate the relaxation of mRNA secondary structures [86]. Also, SLIRP has been linked with the presence of mRNAs in active mitoribosomes [87]. Collectively, mS39 may serve as a platform for the docking of the LRPPRC-SLIRP complex which delivers mitochondrial mRNAs to the ribosome [83]. Then, mS39 facilitates the threading of mRNA through the SSU, and the start codon is selected for the initiation of translation (Figure 4).

LRPPRC-SLIRP complex constitutes a linking bond between transcription and translation and seems to act as a universal activator of translation in mammals. To date, only one translational activator has been found in mammals, that being TACO1, which is involved in the translation of COX1 [88]. In contrast, other eukaryotes are heavily dependent on translational activators for mitochondrial translation. These are regulatory proteins that promote the protein synthesis of specific mitochondrial mRNAs. In yeast, the limited number of protein-encoding mRNAs has allowed the evolution of a specialized mRNA recruitment mechanism where particular protein factors promote the translation of distinct mRNAs according to cellular needs. These translational activators utilize the 5′ UTR to bind and deliver the transcripts to the ribosome [89,90]. Interestingly, yeast’s mRNA exit channel has been remodeled, possibly following the alteration of the SSU rRNA 3′ end which no longer facilitates the start codon selection via the Shine-Dalgarno sequence. Instead, the alignment of the yeast mRNAs might be coordinated by the action of translation activators. The species-specific mS42 and mS43 form a heterodimer located at the edge of the remodeled mRNA exit channel, and along with a series of specific protein elements placed on this site, could act as a platform for translation activators [23].

In plants, the mRNA recruitment mechanism is yet to be determined. However, recent findings point to another ribosomal-specific PPR protein, mS83, which may have a role similar to the protein mammalian mS39. mS83 is positioned at the SSU near the mRNA exit channel and could possibly bind to an AxAAA motif, present in nearly half of the plant’s mitochondrial mRNAs via its PPR moieties [25]. Thus, mS83 could potentially aid the mRNA binding to the mitoribosome, align the mRNA with the SSU and assist in the selection of the start codon from the initiation complex (Figure 4). Nonetheless, although mS83 may constitute a potential recruitment mechanism for a number of the plant’s mRNAs, it is unlikely to be unique, since not all mRNAs contain the AxAAA motif [25]. Rather, it is more likely that a combination of distinct mechanisms is involved in mRNA recruitment in plant mitoribosomes [25]. In compliance with this notion, the existence of translational activators in plant mitochondria is expected, especially if we take into consideration the long 5′ UTRs of plant mitochondrial mRNAs, which can act as a binding site similar to yeast. For instance, proteins such as the recently described RFL8 likely act similar to yeast translation activators in plant mitochondria [91]. In the green algae Chlamydomonas, the initiation mechanism is unknown; however, like in humans, the mRNAs lack 5′ UTRs. Additionally, they are post-transcriptionally polycytidylated at their 3′ UTRs [92]. It is possible that the polycytidylation is important to have translatable mRNA, either by creating a binding platform for trans-acting factors—RNA binding proteins could recognize this cytidine stretch and act as translation activators—or may contribute to mRNA circulation to also promote translation [93].

Altogether, the vast divergence of mitoribosomes across eukaryotes, the presence of novel ribosomal specific proteins, the specialized nature of mitoribosome to decode membrane proteins coupled with the distinct characteristics of mitochondrial mRNAs, had an immense influence in mRNA recruitment, resulting in elegant and highly adapted mechanisms.

### 4.3. Involvement of Mitoribosome Specific Proteins for Protein Binding

Mitoribosomes have followed separate evolutionary routes regarding their RNA contents from being expanded in plants [27] and fungi [23,70], reduced in metazoa [21,22], and kinetoplastids [24], or even fragmented in the green algae Chlamydomonas and in members of the Apicomplexa clade [27,94], to being mostly unchanged in jakobids [30] compared to their bacterial counterparts. In contrast, their protein composition has universally increased across the eukaryotic lineages as a result of various expansions in bacterial proteins as well as the presence of novel species-specific ribosomal proteins, shifting the RNA-protein ratio from the bacterial 2:1 to 1:2 in mammals and 1:6 in kinetoplastids [6].

The novel ribosomal proteins hold a central role in ribosome architecture, assembly, and translation through interactions either with rRNAs (discussed above) or with other proteins. In mammals, for instance, two novel ribosomal proteins mS29 and mS27 form mitochondria-specific bridges between the two subunits. As discussed in 4.1, mS29 is a guanosine 5′-triphosphate (GTP) binding protein located at the head of the small subunit. It interacts with the central protuberance in the LSU creating two bridges with mL46 and mL48 [22]. Another inter-subunit bridge is formed by the novel RP mS27 and the N-terminal extension of bL19m, connecting the bottom of the SSU with the LSU.

Inter-subunit bridges coordinate the relative movements of the subunits, crucial for the active translation. In yeast, the specific connection of mS44 and bL19m contributes to the multiple contact sites between the small and the large subunit of the species. The increased number of inter-subunit bridges present in yeast mitoribosomes compared to humans allows for a more stiff ribosome with the movement of the subunits to be more firm [23].

The presence of novel ribosomal proteins is even more profound in kinetoplastids. Trypanosomal mitoribosomes render an extreme case of divergence, with severely reduced rRNAs and the enrolment of over 120 proteins, half of them being species-specific [24,95]. Notably, the two largest ribosomal proteins mS48 and mS49 (1788 and 1181 aa, respectively) are specific to kinetoplastids. mS48 expands from the lower body of the SSU up to the head interacting with a series of ribosomal proteins (mS51, mS22, mS47, mS60, uS5m, mS59, and mS49), displaying an intrinsic role in the assembly of the SSU and its protein network. mS49 exhibits a similar role through interactions with most of the proteins located at the head of the SSU (uS10m, mS53, mS35, mS55, mS52, and mS50) [24]. A repercussion of the rRNA contraction is the presence of functionally important regions, like the L1 stalk and the central protuberance (CP), which is totally depleted of rRNA. The L1 stalk, through conformational changes, regulates the tRNA positioning and release from the E-site [96,97]. The novel ribosomal proteins mL70, mL74, m91 and the bacterial bL9m that form the L1 stalk compensate for the missing rRNA. Similarly, the CP is solely formed by proteins including the specific mL73, mL82 and mL96, and probably assembles as a module which docks to the LSU at a late intermediate state [95,98,99]. The kinetoplastid’s mitoribosomal exit tunnel is also highly remodeled. The unique r-proteins mL72 and mL109 interact with the conserved uL4m, uL22m, uL23m and uL29m to shape the mature peptide exit channel [95,99].

Another notable example, where novel ribosomal proteins have reshaped the mitoribosome’s architecture, is the evolutionarily distant *Tetrahymena thermophila*, a ciliate protist from the phylum Alveolata. The SSU is characterized by its two protein rich regions, the body extension and the back protuberance [28]. Both regions have a limited rRNA presence, and subsequently they are shaped by multiple protein-protein interactions, many of them attributed to mitoribosomes or proteins only found in ciliate. The body extension is formed by eight proteins (four unique) uS11, mS23, mS26, mS37, mS76, mS77, mS78-NTD and mS79, whereas the back protuberance consists of eleven proteins mS45, mS47, mS78-CTD, mS83–88, mS91 and mS92 (one mitoribosome specific, 10 unique). The two moieties are connected with mS78, the largest ribosomal protein present in ciliate mitoribosomes. In addition, both regions are interconnected with the SSU head, with the body extension to interact with the head through mS23, mS26, mS29, and mS37 and the back protuberance through mS31, mS45, mS47, mS85, mS87, mS88, mS89, mS92, and uS9m [28]. This extensive protein network formed between the head and the body could affect and may limit the movement of the head upon the conformational changes of the SSU during translation elongation [96,100]. Interestingly, half of the proteins synthesized in ciliate mitoribosomes are soluble [28]; however, how mitoribosomes can adapt their mode of synthesis from membrane to matrix proteins is yet to be determined. A notable unique ribosomal protein, mL105, is located near the exit channel and interacts with the uL22m and the uL24m at the solvent side, whereas the mL105-CTD enters the exit tunnel, interacting with bL23m and possibly with the nascent chain. In addition, mL105 possesses a ‘signal peptide-binding domain’ which may constitute some early evidence regarding a putative protein targeting the system in ciliate mitochondria [28]. Finally, an interesting feature of the mRNA entry channel is the reconstitution of uS3 ribosomal proteins out of three separate proteins: mS31m, mS92, and uS3m. The two first, which are nucleus-encoded, interact with the mitochondria-encoded uS3m to form the complete uS3 ribosomal protein, showcasing the significance of the crosstalk between the two genomes as a repercussion of the genetic drift throughout mitochondrial evolution [28].

Collectively, unique mitoribosomal proteins have been recruited, following several evolutionary pressures, to vastly reshape the architecture of eukaryote’s mitoribosomes and take over many elegant mechanisms of the mitoribosomes by establishing a wide protein network.

### 4.4. Specific Processes for Mitoribosome Attachment to Membranes

The majority of proteins still encoded in the mitochondrial genome are hydrophobic membrane proteins, components of the respiratory chain complexes. Due to their hydrophobic nature, these proteins would be challenging to import from the cytosol. This specificity seems to have caused a specialization of the mitochondrial protein translation machinery for the synthesis of hydrophobic membrane proteins [101]. As a consequence of this specialization, it appears that protein synthesis occurs in the mitochondrial matrix at or near the inner membrane. This localization allows for simultaneous membrane insertion of the neo-synthetized peptide during translation [83]. In order to bring the mitochondrial ribosome close to the inner membrane and close to the protein insertion machinery, specific proteins allow the tethering of ribosomes directly to the main component of the Oxidative Assembly (OXA) translocase, the complex responsible for most of the membrane protein insertion of both nuclear and mitochondrially encoded proteins in the mitochondrial inner membrane [102].

The main component of the OXA complex is the Oxa1 protein insertase. It is part of the Oxa1/YidC/Alb3 conserved protein family, which is an evolutionarily conserved protein machinery for the insertion of hydrophobic proteins into bacterial and organellar membranes [103,104,105]. All proteins from this family have five transmembrane domains; YidC proteins can be found in prokaryotes, whereas Alb3 proteins are present in chloroplasts [106,107]. Oxa1 is found in mitochondria, in an orientation with its N-terminal part in the membrane and its C-terminal part located in the mitochondrial matrix. This C-terminal region plays the role of a “Ribosome Binding Domain” (RBD) [108,109]. The RBD is positively charged, but the precise mechanism of interaction between this RBD and mitochondrial ribosome is still unclear. In yeast, it has been shown that cells expressing C-terminal truncated versions of Oxa1 have impaired respiration functions and difficulties to use non-fermentable carbon sources [109]. In addition, yeasts lacking the C-terminal region accumulate improperly inserted mitochondrial proteins in the matrix which should have been inserted in the inner membrane [110]. Oxa1 knock-out mutation leads to a critical deficiency in respiratory complexes III, IV and V, especially for the two subunits of complex IV (cytochrome oxidase c), Cox1 and Cox2 proteins, both mitochondrially encoded. Insertion of critical membrane proteins in the inner membrane, such as the cytochrome b protein, part of the complex III (cytochrome bc1), is also severely reduced.

Mechanisms and proteins involved in mitochondrial ribosome binding with Oxa1 seem to strongly vary between eukaryotes. Nevertheless, these mechanisms are well described for some organisms [111,112].

The link between Oxa1 and the mitochondrial ribosome in yeast is mediated by a non-ribosomal linker protein named Mba1. This protein is able to bind mitochondrial ribosomes near the peptide exit channel, and can then bind the C-terminal domain of Oxa1 [113]. Structural studies using Cryo-EM and Cryo-ET techniques showed that this interaction is stabilized with the help of the rRNA expansion segment 96-ES1 of the large subunit, making a second contact site directly with the inner membrane of mitochondria [114]. This rRNA stabilizing expansion is not found in any other organism for which the mitochondrial ribosome structure has already been resolved.

Unlike in yeast, the structural homolog of Mba1 in mammals, mL45, was identified as an integral part of the large subunit of the mitochondrial ribosome. This protein directly binds on the Oxa1 homolog, the Oxa1L insertase, and positions the peptide exit channel just above it [115]. Recent structural studies show that the mL45 protein is not the only ribosomal protein making contact with Oxa1L. It seems that the C-terminal region of Oxa1L containing the ribosome binding domain makes two additional contact sites, one with uL24m protein, and another with bL28m, uL29m and the large subunit rRNA [112]. These proteins are located near the peptide channel exit and contact the C-terminal extension of Oxa1. The last contact site with rRNA is mediated by a small extension of the C-terminal region of Oxa1L only found in mammals.

In the green algae Chlamydomonas, in situ imaging using cryo-electron tomography suggests that mitoribosomes are all bound to the mitochondrial inner membrane. Moreover, the determination of the Chlamydomonas mitoribosom’s high resolution structure combined with tomography suggests that a specific protein, mL119, located at the peptide channel exit appears to be involved in the interaction between the mitoribosome and Oxa1 to mediate membrane attachment. This protein is not related to the mammals mL45 or the yeast Mba1 [27]. In contrast with green algae, little is known about membrane-tethered mitochondrial ribosome in land plants, but high concentrations of non-ionic detergents are necessary to solubilise ribosomes from Arabidopsis mitochondrial extracts, suggesting that at least part of these ribosomes are tightly bound to the mitochondrial membranes [26]. Still, no homolog proteins of the mammals mL45, the yeast Mba1 or Chlamydomonas mL119 was found encoded in the Arabidopsis genome, implying that land plants might use yet another specific process for membrane attachment [25]. Nonetheless, non-dissociative immunoprecipitation of plant mitochondrial ribosomes co-precipitate Oxa1a [26], and expression of Oxa1a in Oxa1 knock-out yeast cells leads to a partial complementation of the respiratory defects normally present in the mutant [116]. These clues tend to suggest that plant Oxa1a performs similar functions and might have the possibility to bind the ribosome despite the low amino acid identity between the two homolog proteins.

### 4.5. Functions of Mitoribosome Specific Proteins for Ribosome Assembly

Ribosome biogenesis is the crucial process where all the ribosomes components, r-proteins, and rRNAs come together to form the functional translation machinery. This process involves a large number of proteins (maturation factors) and RNAs (such as snoRNAs in cytosolic ribosome maturation) factors that will aid to properly fold, assemble and modify the different elements of the ribosome [117]. Even if some of these maturation factors are conserved from prokaryotes to eukaryotes—these are usually factors involved in the maturation of conserved catalytic areas—the process of ribosome maturation in eukaryotes involves many additional factors, and is much more complex. In eukaryotes, the maturation process is restricted in the nucleus [118], and recent cryo-EM structures have highlighted the intricacy of this process [119,120,121,122,123]. In prokaryotes, biogenesis appears to be quicker and is thus more challenging to capture by structural means, even if some studies do exist [124,125].

In mitochondria, the biogenesis of mitoribosomes is even more complex, as it has to be coordinated between two gene expression compartments [126]. Indeed, the rRNAs, and part of the r-proteins, depending of the organisms, are encoded by the mitochondrial genome and are thus transcribed and processed inside the mitochondria. The rest of the r-proteins are encoded by the nuclear genome and have to be imported into the mitochondria. This is also the case for all the maturation factors involved in mitoribosome biogenesis. Recently, several studies managed to capture mitoribosome assembly intermediates from humans [127,128,129,130,131] and kinetoplastids [95,98,99,132,133], describing different steps and factors involved in the maturation of the large and small mitoribosomal subunits. A portion of these factors were previously described by non-structural means [59,134,135,136], but most of them were described for the first time with these studies, and most importantly, directly observed in action.

Collectively, the studies on the human LSU maturation structurally describe the mode of action of 13 maturation factors mainly involved in the maturation of the PTC. A large portion of these factors are GTPases and helicases involved in the correct folding of the rRNA, or methylases involved in its modification. Most are conserved and have bacterial or eukaryotic homologs; this is the case for GTPB5 and GTBP10 (homologs to ObgE) or are conserved in the same process with other eukaryotes. Thus, DDX28 is mt-LAF2 in kinetoplastids, and most likely Mss116 in yeast [136] and MRM3 corresponds to mt-LAF5-6. One complex that might be conversed across eukaryotes is the ACP block, composed of MALSU1–L0R8F8–mt-ACP in human and mt-Rsfs–L0R8F8–mt-SAF32 in kinetoplastids. It is always present on the large subunit during its maturation and prevents LSU-SSU association, it however does not bind RNA. The complex is composed of MALSU1/mt-Rsfs, a conserved bacterial factor, involved in ribosome hibernation and not in maturation, complemented by the two factors L0R8F8 and mt-ACP, which are specific to mitochondria. Moreover, the redundancy of assembly factors between LSU and SSU, notably mt-ACP and LYR motif proteins, with components of complex I in kinetoplastids, might point towards potential crosstalks between the assembly of ribosomal subunits and respiratory complexes [95,132,137]. On the contrary, the mTERF4 protein involved rRNA stabilization during the PTC maturation appears to be specific to humans.

In kinetoplastids, the detailed structures of maturation complexes of the large subunit present over 30 different maturation factors [95,98,99]. Given that kinetoplastid mitoribosomes are probably among the most divergent mitoribosomes described to date, it is not surprising that so many additional maturation factors were recruited in kinetoplastids (which is also the case for the SSU maturation described later) [24]. Many of the specific maturation factors are RNA binders, but also repurposed mitochondrial proteins, similar to the additional mitochondria-specific r-proteins that were recruited to mitoribosomes [5]. The strong complexity of this mitoribosome, and the extensive number of maturation factors involved, probably makes the biogenesis of this mitoribosome much slower compared to classical ribosomes, which may well have played an important role in the capture of these maturation states. A tryptic of GTPases, mt-EngA, mt-EngB, and mt-Rgb1 (also found in the human LSU intermediates, named MTG1), notably plays an important role in the maturation of the PTC by interacting with the rRNA and locking it in a specific conformation.

Finally, several maturation states were also resolved for the small subunit of *Trypanosoma brucei*, involving in total 39 maturation factors [132,133]. During that process, the SSU goes through drastic remodelling, and many of the assembly factors complement the huge protein shell formed by the already in place r-proteins. Similar to the LSU, a large portion of these factors are involved in the correct folding of the rRNA, notably for the decoding centre. A portion of these maturation factors are KRIPP proteins, kinetoplastids-specific proteins PPR proteins that were previously identified as potential r-proteins [138] that directly interact with single stranded rRNA. For example, KRIPP2 interacts with the 5′ end of the immature rRNA, very similar to OPR mL115 in Chlamydomonas or mL106 in Tetrahymena interactions with mature rRNA.

In summary, mitoribosome assembly involves protein factors conserved with bacteria but also new factors that are similar to mitoribosome r-proteins, either shared between eukaryotes, or specific to certain clades. Many of those factors interact with RNA, notably to promote its correct folding.

## 5. Concluding Remarks

The extraordinary and unexpected diversity of mitochondrial ribosomes began to be recognised less than a decade ago, in particular due to the development of revolutionary cryo-electron microscopy techniques that allowed for the obtaining of high resolution structures of mitoribosomes from a number of distantly related eukaryotes. Initial biochemical analyses suggested that mitoribosomes are protein-rich [36,39,78]. This was corroborated by the first cryo-EM structures obtained with animal models and led to the initial assumption that the recruitment of many novel proteins to mitoribosomes had been driven by the necessity to spatially replace rRNA segments or sub-domains that were lost during evolution. This “structural patching” hypothesis was however contradicted by the observation that yeast and particularly plant mitoribosomes do not have reduced rRNAs, contrary to animals or kinetoplastids, but still have a widely expanded set of ribosomal proteins. Likewise, the bioinformatic and biochemical analysis of mitoribosomes in jakobids, a group of protists with the most bacterial-like mitochondrial genomes and rRNAs, also found that jakobid mitoribosomes are protein rich as compared to their bacterial ancestors [30]. This led to the hypothesis that the last eukaryote common ancestor (LECA) mitoribosome might have already been protein-rich. Indeed, while tens of specific r-proteins were acquired in independent eukaryote clades, phylogeny analyses suggest that LECA might have contained as many as 33 SSU and 46 LSU proteins, thus with a 50% increase of protein numbers as compared to bacterial ribosomes [30]. This raises the question of the nature of the evolutionary forces that might have driven the very early recruitment of many novel proteins to mitoribosomes. One possible explanation might reside in the biochemical environment constituted by the mitochondrial matrix. This compartment is much more basic, with a pH close to 8, than the mitochondrial inter membrane space and the cytosol that has a pH close to 7 [139]. This pH gradient generated by the oxidative phosphorylation (OXPHOS) machinery and required for energy production through the ATP synthase might be deleterious to rRNA stability. Thus, the recruitment of many proteins to the mitoribosome might have been favoured to decrease contacts between rRNAs and a high pH solvent that could result in rRNA hydrolysis. In turn, the occurrence of many additional proteins might have been the evolutionary hint to allow the drift of mitochondrial rRNA genes through either losses or expansions.

Beyond the evolutionary history of mitoribosomes composition, many open questions remain with regard to the diversity of mitoribosomes within the same species, on the regulation of their function, and on their interaction with other gene expression complexes, as well as the assembly machineries of OXPHOS complexes. For instance, it is still unclear if different subpopulations of mitoribosomes, with different protein contents, occur within the same mitochondria. In some eukaryotes, i.e., in plants, hydrophilic proteins are encoded in the mitochondrial genome in addition to the common set of hydrophobic OXPHOS proteins. Does this imply that a subpopulation of mitoribosomes is attached to the inner membrane to synthesize hydrophobic proteins, while another subset of mitoribosomes would be soluble in the matrix for the synthesis of hydrophilic proteins? Answers to such questions are likely to be brought by in situ approaches such as cryo-electron tomography. These analyses are also expected to reveal if some of the additional ribosomal proteins described here play novel or additional functions, e.g., for the interaction of ribosomes with other complexes or for other yet unassigned regulatory processes related or not to mitochondrial translation. Likewise, since mitoribosome protein functions were already related to diseases in humans, e.g., tumorigenesis [140], future studies will also reveal whether dysfunctions of the novel mitochondrial-specific ribosomal proteins described here may result in disorders ranging from decreased agriculture yield to human diseases.

## Data Availability

Not applicable.
